# Dynamic mutual manufacturing and transportation routing service selection for cloud manufacturing with multi-period service-demand matching

**DOI:** 10.7717/peerj-cs.461

**Published:** 2021-04-23

**Authors:** Seyed Ali Sadeghi Aghili, Omid Fatahi Valilai, Alireza Haji, Mohammad Khalilzadeh

**Affiliations:** 1 Department of Industrial Engineering, Science and Research Branch, Islamic Azad University, Tehran, Iran; 2 Department of Mathematics & Logistics, Jacobs University Bremen, Bremen, Bremen, Germany; 3 Department of Industrial Engineering, Sharif University of Technology, Tehran, Tehran, Iran

**Keywords:** Cloud manufacturing, XaaS, Service composition problem, Industry 4.0, Reinforcement learning

## Abstract

Recently, manufacturing firms and logistics service providers have been encouraged to deploy the most recent features of Information Technology (IT) to prevail in the competitive circumstances of manufacturing industries. Industry 4.0 and Cloud manufacturing (CMfg), accompanied by a service-oriented architecture model, have been regarded as renowned approaches to enable and facilitate the transition of conventional manufacturing business models into more efficient and productive ones. Furthermore, there is an aptness among the manufacturing and logistics businesses as service providers to synergize and cut down the investment and operational costs via sharing logistics fleet and production facilities in the form of outsourcing and consequently increase their profitability. Therefore, due to the Everything as a Service (XaaS) paradigm, efficient service composition is known to be a remarkable issue in the cloud manufacturing paradigm. This issue is challenging due to the service composition problem’s large size and complicated computational characteristics. This paper has focused on the considerable number of continually received service requests, which must be prioritized and handled in the minimum possible time while fulfilling the Quality of Service (QoS) parameters. Considering the NP-hard nature and dynamicity of the allocation problem in the Cloud composition problem, heuristic and metaheuristic solving approaches are strongly preferred to obtain optimal or nearly optimal solutions. This study has presented an innovative, time-efficient approach for mutual manufacturing and logistical service composition with the QoS considerations. The method presented in this paper is highly competent in solving large-scale service composition problems time-efficiently while satisfying the optimality gap. A sample dataset has been synthesized to evaluate the outcomes of the developed model compared to earlier research studies. The results show the proposed algorithm can be applied to fulfill the dynamic behavior of manufacturing and logistics service composition due to its efficiency in solving time. The paper has embedded the relation of task and logistic services for cloud service composition in solving algorithm and enhanced the efficiency of resulted matched services. Moreover, considering the possibility of arrival of new services and demands into cloud, the proposed algorithm adapts the service composition algorithm.

## Introduction

Recently, rivalry economic circumstances have compelled manufacturing industries to take advantage of modern Information Technology (IT) to reinforce their manufacturing processes’ capabilities and enhance their profitability (Kang et al., 2016; Lasi et al., 2014). Enormous research studies have tackled this issue to improve and optimize the operations’ efficiency and productivity. Particularly, considering the emergence of new manufacturing paradigms such as Cloud manufacturing and Industry 4.0 has resulted in virtualized factories and outsourcing mechanisms (Tao et al., 2017; Wang et al., 2019; Li et al., 2010). These paradigms are realized in acquiring services, which have shaped the new business models of manufacturing systems and transformed them from process/product-oriented systems to service-oriented ones (Saldivar et al., 2015; Delaram & Valilai, 2018; Wu et al., 2013). In addition, pooling manufacturing and logistics capabilities has been found to reduce operational and investment expenses and hence increased profit (Kang et al., 2016).

In recent years, Industry 4.0 and Cloud manufacturing, accompanied by the Service Oriented Architecture (SOA), have been profoundly regarded as essential means to facilitate the transition from conventional manufacturing system to efficient, modern, and decentralized system (Saldivar et al., 2015; Valilai & Houshmand, 2015; Tao & Qi, 2019). The fundamental methodology of service provision, which Cloud manufacturing works upon, is that the entire supply network is deemed the aggregation of modules and blocks that can be outsourced and provided in perhaps diversely located service points and fulfilled by different service providers. These service providers will then collaborate to fulfill an order by collaborating their resources (Tao et al., 2017; Zhu et al., 2020c). This service composition approach for fulfilling complicated orders is believed to result in higher quality, productivity, and versatility (Lasi et al., 2014; Saldivar et al., 2015; Tao & Qi, 2019).

Cloud manufacturing, is mainly referred to and elaborated with the help of the Everything as a Service (XaaS) concept. This concept persuades service providers to deploy the most state-of-the-art high-tech and cybernetics, namely Internet of Things (IoT) and Blockchain (Zhu et al., 2020c; Li, Barenji & Huang, 2018; Zhu et al., 2020b). On the other hand, this enables clients to access a wide range of services to select from and compose them to fit best their requirements (Saldivar et al., 2015; Qi & Tao, 2019). Since this service composition problem is discussed as NP-hard (Delaram & Valilai, 2018), heuristic and metaheuristic methods are strongly preferred to obtain the optimal or nearly optimal solutions in a short solving time. However, considering the dynamic characteristics of services, the challenge will grow. This study is dedicated to introducing a real-time service assignment framework, considering the Quality of Services (QoS) as a top priority, also considering logistical issues besides manufacturing services. The impulsive variations in the inputs, such as the quantity of the operations/demands and the Quality of Services, or any unforeseen hindrances due to the manufacturing facilities, are considered dynamic (Wang et al., 2020; Zhou & Yao, 2017). The dynamic nature of the problem can lead to complications and imperfections in the Cloud manufacturing service composition. This paper significantly focuses on handling the new service requests in manufacturing and logistics service composition problem in the pre-specified time-intervals that occur while solving the problem. Furthermore, this article has studied the latest relevant research studies in the Cloud manufacturing service-composition problem to justify the research gap. In addition, the simultaneous logistical and manufacturing service-composition has been considered in this research. The paper has proposed an innovative solution for solving time with particular attention to large-sized problems and also the necessity for re-scheduling the newly received service requests.

## Literature review

The paper first studies the most recent dominant research studies, which focus on Cloud manufacturing service composition in large-scale problems focusing on solving efficiency. Aghamohammadzade and Valilai tackled the service composition problem via a decentralized peer-to-peer strategy in the Cloud manufacturing environment utilizing Blockchain named Block-SC. In their approach, the problem is broken into a number of sub-problems, each including a part of the total service required and to be solved by a different solver. Solvers are supposed to find the fittest solution, the one with the minimum global cost and service time (Aghamohammadzadeh & Fatahi Valilai, 2020). In addition, one key thing in this regard is to curb the number of customers lost during the procedure due to the method’s restrictions. This issue is of great importance since failing to respond to and satisfying the customers is equivalent to losing eminence and prestige in the marketplace. Although they considered the Cloud manufacturing environment’s dynamicity, solved the problem at an acceptable time, and tried to localize the service providers, they did not consider the QoS and the transportation service.

Liang et al. introduced PD-DQN—a Deep Reinforcement Learning (DRL) algorithm—which utilizes the basic Deep Q-Network (DQN) in the Cloud manufacturing environment, together with the dueling architecture, as well as the prioritized replay mechanism (Liang et al., 2021). In their approach, the Quality of Service and transportation are also regarded. Moreover, it is capable of learning optimal and nearly optimal solutions with no need for any foreknowledge or adjustment of hyper-parameters. In their work, they modeled the service-composition process into a Markovian Process. The results proved that the algorithm is highly efficient, effective, and flexible to dynamicity and the problem’s size. In conclusion, they focused on Cloud Manufacturing Service Composition (CMfg-SC), and the dynamic nature of the problem, logistics, and QoS was considered in their proposed method. Besides, they measured the performance of their algorithm. However, no runtime evaluation was conducted.

Wang et al. presented an approach under Service Composition Exception Handling Adaptive Adjustment (SCEHAA) (Wang et al., 2020). They believed that service composition in Cloud manufacturing has some imperfections in dealing with unexpected, irregular situations such as machinery break down, decrease or increase in service requirement, changes in the quality of provided services, and so, which are interpreted to dynamicity. They innovated a method utilizing the improved Ant Colony Optimization Algorithm (ACO), which is claimed to successfully deal with all these exceptional circumstances by adjusting time boundaries and operational and non-operational costs, as well as observing the Quality of Service in a manner that still optimal solutions are achieved. The adjustment process is committed to deadlines, and the algorithm is of high convergence. Nevertheless, they considered the productivity, logistic service, and dynamicity of the problem and carried out experiments that showed their algorithm needs fewer iterations than opponent algorithms. There was no execution time evaluation included.

Zhu et al. (2020a) introduced a modified Artificial Bee Colony (ABC) algorithm with enhancements on the bee exploring mechanism called Multiple Improvement Strategies based Artificial Bee Colony algorithm (MISABC). They believed that the ABC algorithm was convenient to apply and of few parameters with acceptable results, amply utilized in Cloud manufacturing service composition. However, in large-sized problems, there have been complications and deficiencies. These deficiencies were tackled via approaches namely Differential Evolution (DES) that accelerated the algorithm’s convergence, Oscillation Strategy with classical Trigonometric Factor (TFOS), Different Dimensional Variation Learning Strategy (DDVLS) along with Gaussian Distribution Strategy (GDS) to make a better chance to achieve a global optimum. Nonetheless, they had conducted experiments that showed their algorithm solutions were mostly better than Particle Swarm Optimization (PSO), classic Artificial Bee Colony (ABC), and Differential Evolution (DE); they neither considered logistics nor QoS in their research. Also, there was no record of runtime evaluation found.

Wang et al. (2018) optimized the Cloud manufacturing service-composition using a Two-stage Biogeography-Based Optimization algorithm (TBBO), characterized by being immediate, quality-concerned, and suited to handle a particular dynamic condition. It works as follows. First, the service composition problem is solved, and the optimal solution is attained (stage one). When the service composition system faces an unanticipated demand, the problem is resolved according to the new requirements, so the service is re-composed (considered stage two). In order to fulfill the deadlines for the current demands, as well as the new ones, they propose two different methods. The first one is here briefly referred to as Vertical Collaboration, and the second one is called Speed up Strategy.

In a conventional Cloud manufacturing service-composition, an operation is accomplished via horizontal cooperation between upstream and downstream services in a supply chain, and each service is assigned to only one operation. On the opposite, according to the vertical collaboration method, each sub-operation is allocated to a number of services that enable saving performing time. In the Speed Selection-based Recomposition (SSR) method, it is supposed that the machinery is potentially capable of working faster or handling more operations than are scheduled for. This extra capacity is counted on in re-composition to fulfill the newly added tasks. Although they considered the dynamic nature of Cloud manufacturing, the logistics were still not considered, and there was no runtime evaluation included.

Liu et al. (2013) improvised a Multi-Composition for Each Task (MCET). They joined the unavailing compound system to form a consolidated system to enable performing multiple-operation manufacturing tasks. This system guarantees the global quality to the required level. A Hybrid-Operator-based Matrix Coded Genetic Algorithm (HO-MCGA) is exploited in their approach, which achieved more enhanced fitness results compared to MCGA using simplex-operator. They also considered neither the logistics nor the dynamicity of the problem. However, according to their results, the fitter solutions were achieved; it was mostly slower than MCGA (up to 30% in some cases).

Liu et al. (2016) were among the first researchers who addressed multiple task service allocation and scheduling in the cloud environment. A distinct point in their research is that dynamicity with reference to abrupt changes such as service accessibility was regarded. Moreover, resource and time-sharing among individual service providers, as well as logistics, were taken into consideration. Even though they developed such a great model that considered all the major issues like logistic services, QoS, and the problem's dynamic nature, there is no report on run time evaluation. They should have considered at least one algorithm to solve a problem to verify the performance of the developed model.

Que et al. proposed an innovative Manufacturers to Users (M2U) model for the cloud environment to provide a solution to the core Manufacturing Service Composition Optimal Selection (MSCOS) problem, which exploited a modified flexible Information Entropy Immune Genetic Algorithm (IEIGA) (Que et al., 2018). This model that was meant to apply to process industries uses some essential Quality of Service indices—time, reliability, cost—each was associated with an influence coefficient yielded by a combination assignment method. The experiments verified the presented method of a higher quality of performance regarding large-sized MSCOS problems compared to the Standard Genetic Algorithm (SGA) and Immune Genetic Algorithm (IGA). However, the numerical results showed that IEIGA had a better performance considering the results’ quality; their conducted results showed that the IEIGA needs more time than SGA (Almost five times more). Again, logistics was not considered.

Zhou & Yao (2017) presented a context-aware Artificial Bee Colony (caABC) algorithm based on service characteristics in the cloud environment enhanced by the Differential Evolution so-called DE-caABC, which is intended to improve the exploration method of the algorithm. They tackled prevailing deficiencies in CMfg service-composition methods, which can be summarized as lack of flexibility regarding dynamic situations such as newly-acquired services or operations, service failures, changes in Quality of Service and so, that may happen during the scheduling and assignment process, complications in handling large-sized problems, and the service domain features that can impact the performance of service-composition like correlation, similarity and so. Nevertheless, they considered dynamic QoS, and results showed that the quality of their proposed method’s solution overcomes GA, DE, ACO, ABC, and PSO in most cases. The conducted runtime evaluation showed that it needed almost twice as much time in comparison with pure GA. What is more, transportation services were not considered.

Zheng, Feng & Tan (2016) tried a consolidated service selection method that enables clients to receive optimal services. They developed a model of Cloud manufacturing based on the Quality of Service preference. Knowing that the Quality of Services available in CMfg is varied and fuzzy, a model using Fuzzy Theory as its basis is produced to determine the Quality of Service. The Particle Swarm Optimization (PSO) algorithm is exploited to attain the optimal solution. However, they conducted a model that considered QoS preferences and transportation; they did not consider the problem’s dynamic nature. They also conducted a runtime and fitness evaluation, but no comparison with other popular algorithms was presented.

Tao et al. described Service Composition Optimal-Selection (SCOS) as NP-hard, erratic, and dynamic, which needed careful consideration in CMfg (Tao et al., 2013). Therefore, they proposed an alternative algorithm, called Full Connection-based Parallel Adaptive Chaos Optimization with Reflex Migration (FC-PACO-RM). The latter was deployed to upgrade the quality and efficiency of the solution. Moreover, roulette wheel selection and adaptive chaos optimization were utilized as their searching method, together with Full-connection Parallelization in the Island model. In addition, full connection topology-based on coarse-grained parallelization and MPI collective communication was utilized to enhance their search efficiency. While they focused on SCOS and measured their algorithm’s performance, the logistics were not considered. Moreover, even though the algorithm showed a better fitness value comparing to GA, CGA, and CO, it did not outweigh GA in runtime.

Akbaripour et al. proposed new Mixed-Integer Programming (MIP) models to solve service composition problem in cloud manufacturing regarding QoS and Transportation. Their model consists of four basic building blocks, namely sequential, parallel, loop, and selective. They tried different transportation scenarios and concluded that a combination of Hub-and-Spoke and direct transportations yields the optimum time and cost solution. According to their study, including transportation leads to a significant increase in time and cost (Akbaripour et al., 2018). They found optimal solutions with acceptable runtime for small to medium-sized problems. However, they did not investigate any large-sized problems. What is more, according to their paper, the dynamic nature of the CMfg problem was not considered in their research.

Zhou et al. proposed an algorithm to solve the logistics service scheduling problem with the main focus of reducing the average task delivery time. They simulated four scenarios including, Total Time-based Logistic Scheduling (TTLS), Fastest Logistics Strategy (FLS), Nearest Logistics Strategy (NLS), and Random Logistics Strategy (RLS). Their study revealed that TTLS obtains the shortest time, while NLS and RLS have almost similar results, and FLS results in the worst delivery time (Zhou, Zhang & Fang, 2020). Nonetheless, they presented a method to shorten the delivery time, cost optimization and QoS considerations were not of their concern. Moreover, there is no evidence of comparing their model’s performance against any of the well-known algorithms like GA.

Lartigau et al. (2015) proposed an enhanced ABC algorithm. Their study included the impact of transportation and QoS. They defined a fitness function that consisted of the price, time, availability, maintainability, reliability, and ecological impact of the production, giving each one a degree of importance. They developed a comprehensive model with realistic concerns and claimed that their enhanced ABC has better runtime than PSO or GA. They reached the optimal solution for small-sized problems; however, only nearby solutions could be obtained for bigger problems. They suggested tuning the algorithm’s parameters using Artificial Neural Networks (ANN) to improve the quality of the solution. However, since the tuning runtime was not included in their study, it is difficult to compare the runtime’s overall performances. They utilized Java Genetic Algorithms Package (JGAP), but they did not state what languages were used to implement the two other algorithms. As different programming languages’ performance can affect the runtime, it can not be concluded that their enhanced ABC is always better than GA (Lartigau et al., 2015).

Table 1 also compares the aforementioned research studies. As observed, the focus on considering logistics and manufacturing services with their dynamic behavior in multi-window planning has not been considered in the literature. This issue will be the focus of this paper. The paper has proposed a framework to help the service composition problem while fulfilling the dynamic service or demand behavior.

**Table 1:** Research studies comparison.

Research	Contribution	Large Scale Service Selection Method	Transportation services	Problem Dynamicity	QoS
Aghamohammadzadeh & Fatahi Valilai (2020)	A novel Cloud manufacturing service composition platform enabled by Blockchain technology	Block-chain based method called Block-SC		✓	
Liang et al. (2021)	Logistics-involved QoS-aware service composition in Cloud manufacturing with deep reinforcement learning	Deep Reinforcement Learning algorithm called PD-DQN	✓	✓	C/P/R/T
Wang et al. (2020)	An effective adaptive adjustment method for service composition exception handling in Cloud manufacturing	improved ant colony optimization algorithm called SCEHAA	✓	✓	C/P/T
Zhu et al. (2020a)	A Novel Service Composition Algorithm for Cloud-Based Manufacturing Environment	Multiple Improvement Strategies based Artificial Bee Colony algorithm (MISABC)		✓	
Wang et al. (2018)	Urgent task-aware Cloud manufacturing service composition using two-stage biogeography-based optimization	A two-stage biogeography-based optimization algorithm (TBBO)		✓	C/R/T
Liu et al. (2013)	Study on multi-task-oriented services composition and optimization with the ‘Multi-Composition for Each Task’ pattern in Cloud manufacturing systems	Hybrid-Operator based Matrix Coded Genetic Algorithm (HO-MCGA)			C/T
Liu et al. (2016)	An Extensible Model for Multitask-Oriented Service Composition and Scheduling in Cloud manufacturing		✓		C/R/T
Que et al. (2018)	Improved adaptive immune genetic algorithm for optimal QoS-aware service composition selection in Cloud manufacturing	information entropy immune genetic algorithm (IEIGA)		✓	C/R/T
Zhou & Yao (2017)	DE-caABC: differential evolution enhanced context-aware artificial bee colony algorithm for service composition and optimal selection in Cloud manufacturing	differential evolution enhanced context-aware artificial bee colony algorithm (DE-caABC)		✓	A/P/T/W
Zheng, Feng & Tan (2016)	A fuzzy QoS-aware resource service selection considering design preference in Cloud manufacturing system	Particle Swarm Optimization	✓		A/C/R/T/W
Tao et al. (2013)	FC-PACO-RM: A parallel method for service composition optimal-selection in Cloud manufacturing system	Adaptive Chaos Optimization and Full-Connection Parallelization in the Island model		✓	C/E/M/R/S/T/Z
Akbaripour et al. (2018)	Cloud manufacturing service selection optimization and scheduling with transportation considerations: mixed-integer programming models	Mixed-Integer Programming	✓	✓	C/Q/T
Zhou, Zhang & Fang (2020)	Logistics service scheduling with manufacturing provider selection in cloud manufacturing		✓		
Lartigau et al. (2015)	Cloud manufacturing service composition based on QoS with geo-perspective transportation using an improved Artificial Bee Colony optimisation algorithm	Artificial Bee Colony Algorithm	✓		A/E/C/M/R/T
This Research	Dynamic Mutual Manufacturing and Transportation Routing service selection for Cloud manufacturing with Multi-Period Service-Demand matching	Genetic Algorithm	✓	✓	C/P/Q/T

**Note:**

A: Service Availability, C: Cost, E: Energy, I: Cooperation intensity, M: Maintainability, P: Performance, Q: Quality, R: Reliability, S: Function Similarity, T: Time, U: Usability, W: Reputation, X: Credibility, Y: Composability, Z: Trust

## The presented model for dynamic mutual logistic and manufacturing service composition

As previously mentioned, the Cloud manufacturing paradigm provides a solution to coordinate non-centralized resources and services through the XaaS approach. This paradigm intends to attain better performance in terms of productivity and efficiency. Nonetheless, minimizing the matching time of the logistics and manufacturing parts of the system—to obtain a coordinated system with the highest possible quality—is a significant challenge. Furthermore, the problem’s dynamic nature must be considered as a tremendously challenging issue, which means a new task could be added to the existing ones at any time during the process that may lead to resolving the remaining part of the scheduling problem.

Although extensive research has been conducted on single and multiple task scheduling problems in cloud computing, the fact that distinguishes Cloud Manufacturing from Cloud Computing is that both logistic and manufacturing planning have to be considered at the same time, which adds to the complexity of the problem. The other aspect that makes the present study distinct is that—unlike the prior studies—it provides a mathematical model for the Cloud manufacturing service composition problem to solve this scheduling problem. The service composition problem in Cloud Manufacturing is recognized by previous researchers as strongly NP-Hard (Delaram & Valilai, 2018; Que et al., 2018). Moreover, finding the appropriate solution will be a great challenge regarding the Cloud manufacturing system’s large size. The contribution of this study is to tackle both logistic and manufacturing services allocation exploiting a heuristic method to solve the service composition problem applied to real dynamic large-scale problems.

Considering the complex nature of this assignment-scheduling problem, firstly, the problem was simplified. Therefore, an appropriate choice seemed to be an extended version of the Assignment Problem (AP). In this model, the (manufacturing) services were assigned to fulfill the sub-operations of each operation. Also, the transportation services were programmed to carry semi-manufactured parts between service points located in different places. All the unfinished services and new service demands were dealt with and re-scheduled at the pre-specified scheduling horizons. “Mathematical Model” is dedicated to an overview of Delaram and Valilai’s proposed model (Delaram & Valilai, 2018), on which this research will extend. Their presented model considers only a one-time window for service composition. It assumes the system’s static behavior and can not react to the dynamic arrival or modifications of services and demands. The paper will extend the model for enabling the fulfillment of dynamic behavior of service and demands. Afterward, in “The Presented Algorithm for Solving the Model Considering Runtime and Dynamicity”, the proposed algorithm will be explained.

### Mathematical model

Based on extending the model proposed by Delaram & Valilai (2018), The objective function is primarily defined as a cost-minimization function, which includes operational and logistical costs. The acceptable solution could be considered the one that fulfills production needs as well as the QoS standard criteria.

Assumedly, there are S service points to perform different operations to complete the sub-operations of an operation. Index lϵ{1,2,3,…,L} indicates operations performed at each service point, given that n is the number of the operations, which are accessible at a particular instant in the Cloud manufacturing environment, and $O_{n}\in \left\{O_{1},\;\ldots ,\;O_{n},\;\ldots ,\;O_{N}\right\}\;represents\;$the $n_{th}\;$received operation out of total N operations. Each operation should be decomposed into sub-operations to be scheduled and carried out in the Cloud manufacturing platform.

In Cloud manufacturing, two different situations are presumable. In the first state, since two following sub-operations of an operation are accomplished at the same service point or station, no transportation occurs. While in the second state, logistical scheduling is required, as the sub-operations of the operation have to be performed at different locations. However, this assumption is not required in cloud computing, which leads to a notable difference between the two paradigms of Cloud manufacturing and cloud computing. As stated earlier, the objective function—cost-minimization function—is composed of operational costs and logistical costs. Here the operational costs are assumed to be only influenced by the operation performing time, where the distance between the service points exclusively determines the logistical costs. Table 2 exhibits the description of variables and parameters:

**Table 2:** Denotation of the parameters and variables.

Indexes	Description
l	Sub operation number, $l\in L_{n}$
m	Transportation service number, m∈M
n	Operation number, n∈N
s	service point number, s∈S
**Parameters**	**Description**
$TC_{m\left(s,s^{\prime }\right)}$	transportation service m’s cost between service point s and $s^{\prime }$, $TC_{m\left(s,\;s^{\prime }\right)}=\;tc_{m}d_{ss^{\prime }}$ ($)
$PC_{lns}$	the performance cost of subtask $SO_{ln}$ in service point s, ($)
$d_{ss^{\prime }}$	Distance between service points s and $s^{\prime }$ (mile)
$t_{lns}$	Operation performing time $SO_{ln}$ in service point s (hour)
$q_{s}$	Service quality of service point s (%)
$opc_{lns}$	The operation performing cost $SO_{ln}\;$in service point s ($ per hour)
$lc_{m}$	The cost of transportation service m ($ per mile)
$SO_{ln}$	$l_{th}$ sub-operation of the $n_{th}$ operation
$O_{k}$	$n_{th}$ operation
$V_{ln}$	a binary parameter, $V_{ln}=\;$1, if operation n needs sub-operationl, otherwise $V_{ln}=\;$0.
**Variables**	**Description**
$\sigma_{lns}$	a binary variable, $\sigma_{lns}=$ 1, if sub-operation l for operation n in service point s is to be fulfilled, otherwise $\sigma_{lns}=\;$0.
$\rho_{m\left(s,\;s^{\prime }\right)}^{n\left(l,\;\;l+1\right)}$	a binary variable, $\rho_{m\left(s,\;s^{\prime }\right)}^{n\left(l,\;\;l+1\right)}=$ 1, if transportation service m between two service points s and s′ for proceeding from sub-operation l to l+1 for operation n is needed, otherwise $\rho_{m\left(s,\;s^{\prime }\right)}^{n\left(l,\;\;l+1\right)}=\;$ 0.

(1) $$\min\omega =\;\underset{n\in N}{\sum }\underset{l\;\in L_{n}}{\sum }\underset{s\in S}{\sum }PC_{lns}\gamma_{lns}+\;\underset{m\in M}{\sum }\underset{n\in N}{\sum }\underset{\begin{array}{c} s,\;s^{\prime }\in S\\ s\neq s^{\prime } \end{array}}{\sum }\underset{l\in L_{n}}{\sum }TC_{m\left(s,\;s^{\prime }\right)}\rho_{m\left(s,\;s^{\prime }\right)}^{n\left(l,\;l+1\right)}$$

(2) $$\sum_{s\in S}^{s.t.}\sigma_{lns}=\;V_{ln}\;\forall l\;\in L_{n},\;\forall n\in N$$

(3) $$\sigma_{lns}+\;\sigma_{\left(l+1\right)ns^{\prime }}\;-1\;\leq \sum_{m\in M}\rho_{m\left(s,\;s^{\prime }\right)}^{n\left(l,\;l+1\right)}\;\forall l\;\in L_{n},\;\forall n\in N,\;\forall s,\;s^{\prime }\in S$$

(4) $$\rho_{m\left(s,\;s^{\prime }\right)}^{n\left(l,\;l+1\right)},\;\sigma_{lns}=\left\{0,\;1\right\}\;\forall l\;\in L_{n},\;\forall n\in N,\;\forall m\in M,\;\forall s,\;s^{\prime }\in S$$

the above equations demonstrate the model.

Equation (1) models the cost minimization function (objective function), wherein the first expression represents the operational performing costs, while the second part corresponds to the logistical costs. Equation (2) guarantees that each sub-operation is carried out only in one service point and only once if the $l_{th}$ sub-operation is required for operation n. Equation (3) constrains the logistic service to only one service if any logistic service is required between two service points to fulfill two following sub-operations. Equation (4) assures that all variables are binary.

### The presented algorithm for solving the model considering runtime and dynamicity

This paper suggests a customized genetic algorithm approach considering the NP-Hardness of this problem to deal with the challenge of providing a time-efficient solution.

#### Algorithm’s inputs

**Figure 1:**
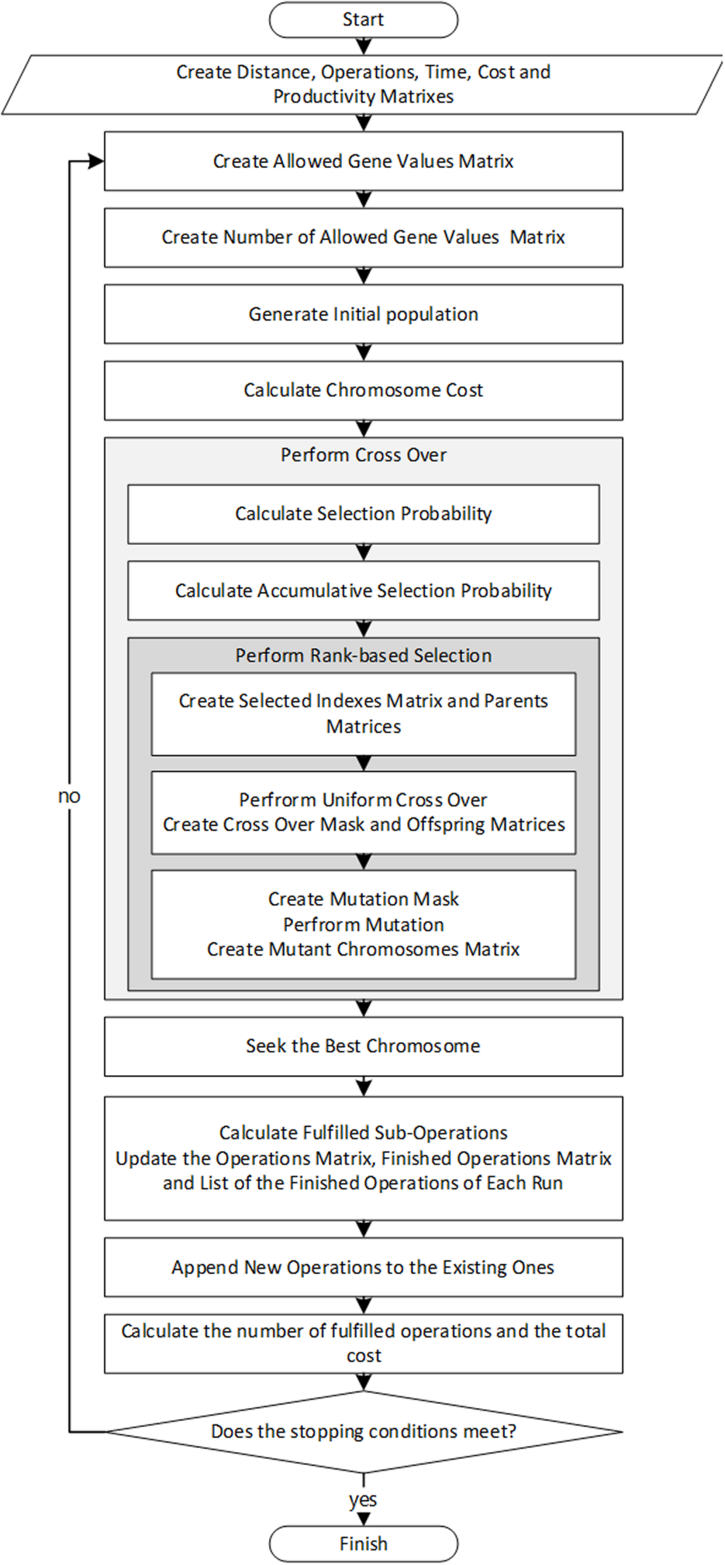
The flowchart of the algorithm.

In “Distance Matrix” to “Number of Allowed Gene Values Matrix”, the input parameters suited to the problem nature are described; further on, the proposed algorithm to solve the problem will be presented in “Solution Algorithm”. Also, Fig. 1 depicts the Algorithm’s flowchart to simplify following the proposed algorithm’s steps.

##### Distance matrix

To model large-sized problems, service points are assumingly distributed/located in a great many cities. The Distance Matrix is filled with the distances between pairs of cities defined as service points. However, the Euclidean distances between the cities are simply taken as the distances between cities. They are calculated, having the cities' coordinates, and put in the Distance Matrix.

##### Operations matrix

Sub-operations form the rows of this matrix, while the operations form the columns. An element of the matrix is one only if the operation needs the corresponding sub-operation; otherwise, it is zero.

##### Time and cost matrixes

In the two matrixes, rows stand for sub-operations, while columns stand for cities (service points). The Time Matrix is filled with the performing time for each sub-operation in the corresponding city, and the Cost Matrix consists of the performing cost of each operation in each city. Nonetheless, if a city does not supply a service(sub-operation), both performing time and cost are presumed infinity.

##### Productivity matrix

The Productivity Matrix is a one-row matrix whose columns represent the cities. Each element of this matrix refers to the Quality of Service offered in each city stated as a percentage.

##### Operation/sub-operation matrix

The Operation/Sub-operation Matrix is one of the essentials of the model. In this matrix, the column numbers refer to the operation numbers and the number of the sub-operation of the respective operation. Hence, the operation number is calculated from formula 5, in which the column number is divided by the maximum number of the sub-operations; after subtracting a very small number from the result, this figure is rounded up.

(5) $$operation\;number\;=\left\lceil \;\frac{column\;number}{maximum\;number\;of\;the\;sub\;operations}-\;0.001\right\rceil$$

For example, if each operation has a maximum number of ten sub-operations, then for the fifteenth column, it results in $\frac{15}{10}-\;0.001=2$.

That indicates this sub-operation belongs to the second operation.

Moreover, as displayed in Eq. (6), the sub-operation number of each operation is (a) equal to the remainder of division (the column number to the maximum number of sub-operations) if it is not zero, (b) equal to the maximum number of sub-operations where the remainder is zero.

(6) $$suboperationnumber=\{\begin{array}{c} mod(\frac{columnindex}{maximumnumberofsuboperations})(a)\\ maximumnumberofsuboperations(b) \end{array}$$

Considering the aforementioned example:

$mod\left(\frac{15}{10}\right)=5$

Here are two more examples: the number of the operations and sub-operations for columns 73 and 20 were calculated as follows.

73/10−0.001=7

$mod\left(\frac{73}{10}\right)=3$

20/10−0.001=2

$mod\left(\frac{20}{10}\right)=0\;\to 10$

##### Allowed gene values matrix

This is a matrix with the maximum number of the cities (service points) as the number of rows and the columns representing operation/sub-operations. As the first step to form this matrix, a zero matrix with the appropriate dimensions is built. Secondly, the (index) number of every city that offers the required service/operation is inserted into the matrix, initiating with the first row in an ascending manner. Apparently, if some cities do not provide a specific sub-operation, the corresponding operation/sub-operation rows will remain zero.

##### Number of allowed gene values matrix

**Figure 2:**
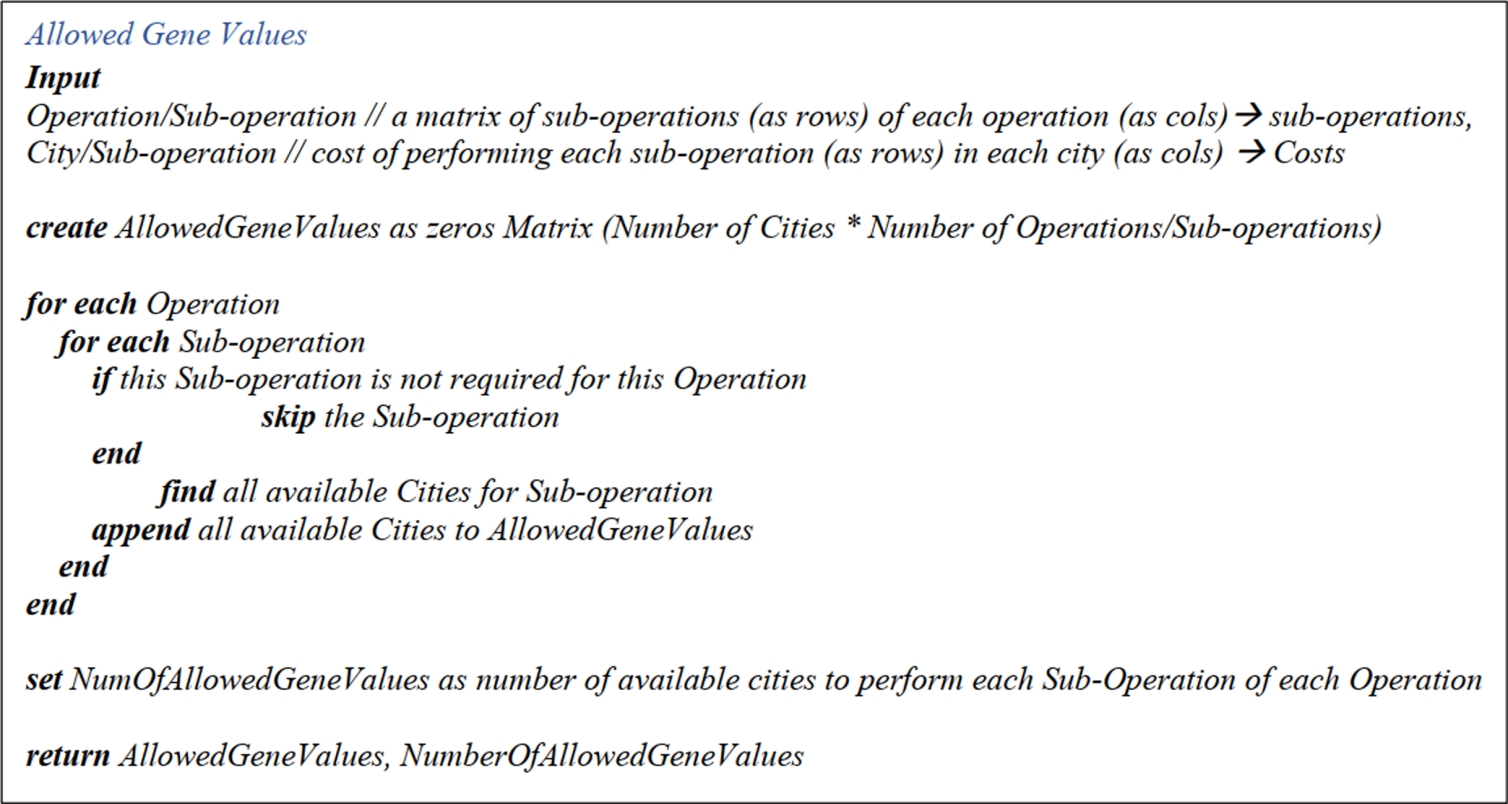
Allowed gene values and number of allowed gene values pseudo-code.

It is also a one-row matrix whose columns denote the operation/sub-operations and contains the number of cities where an operation/sub-operation can be performed. The pseudo-codes for “Allowed Gene Values Matrix” and “Number of Allowed Gene Values Matrix” can be found in Fig. 2.

#### Solution algorithm

As stated earlier, one of the major concerns of the present study is to tackle the dynamic nature of service composition allocation in the Cloud manufacturing environment. Unanticipated situations such as unforeseen changes in the quality or quantity of the required services, machinery non-fulfillment, or so, that may happen during the algorithm runtime, can seriously impact the scheduled services. In this study, an iterative approach is featured to address one of the dynamic situations: newly required services.

In this approach, the problem is reconsidered in pre-specified time intervals, and all the unfinished operations are re-scheduled, as well as recently added demands.

Therefore, after steps “Distance Matrix” to “Operation/Sub-operation Matrix” are executed, the following steps will be repeated for every run until the stopping condition (a particular number of runs or pre-specified runtime) is fulfilled.

**Figure 3:**
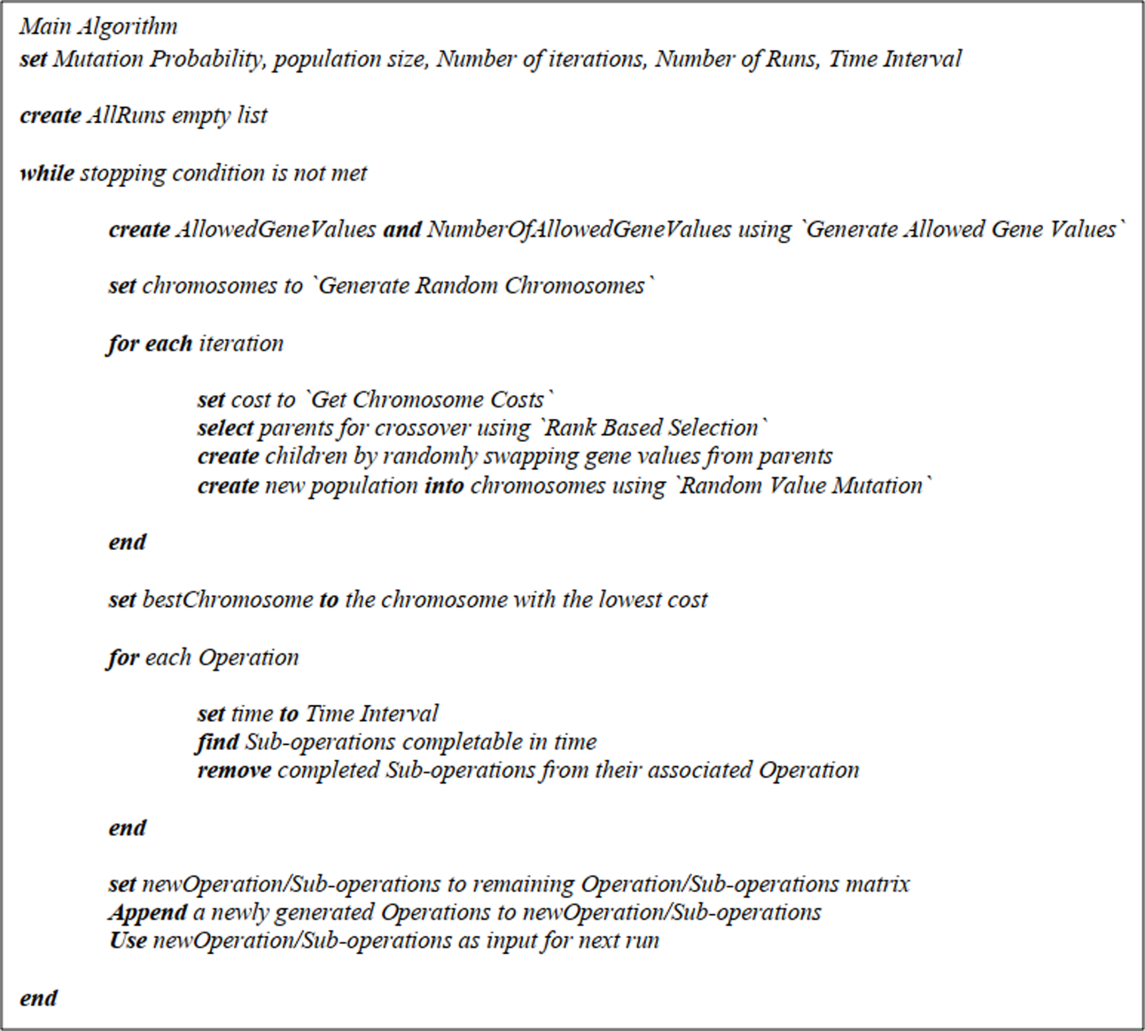
The main algorithm pseudo-code.

This section is devoted to different parts of the main algorithm and the according pseudo-codes. The main pseudo-code could be found in Fig. 3.

**Generating the Allowed Gene Values and Number of Allowed Gene Values Matrixes**

According to “Allowed Gene Values Matrix” and “Number of Allowed Gene Values Matrix”, the Allowed Gene Values Matrix and the Number of Allowed Gene Values Matrix are generated considering the incomplete operations. Then the Genetic Algorithm will be executed as demonstrated in “Initial Population Generation” to “Mutation, Mutation Mask, Mutant Chromosomes Matrix”.

##### Initial population generation

The initial population generation matrix dimensions are as follows: the size of the initial population (chromosomes) is taken as the number of rows, and the number of the operations multiplied by the maximum number of sub-operations (operation/sub-operation) as the number of columns. First, a zero matrix with the above dimensions is generated.

**Figure 4:**
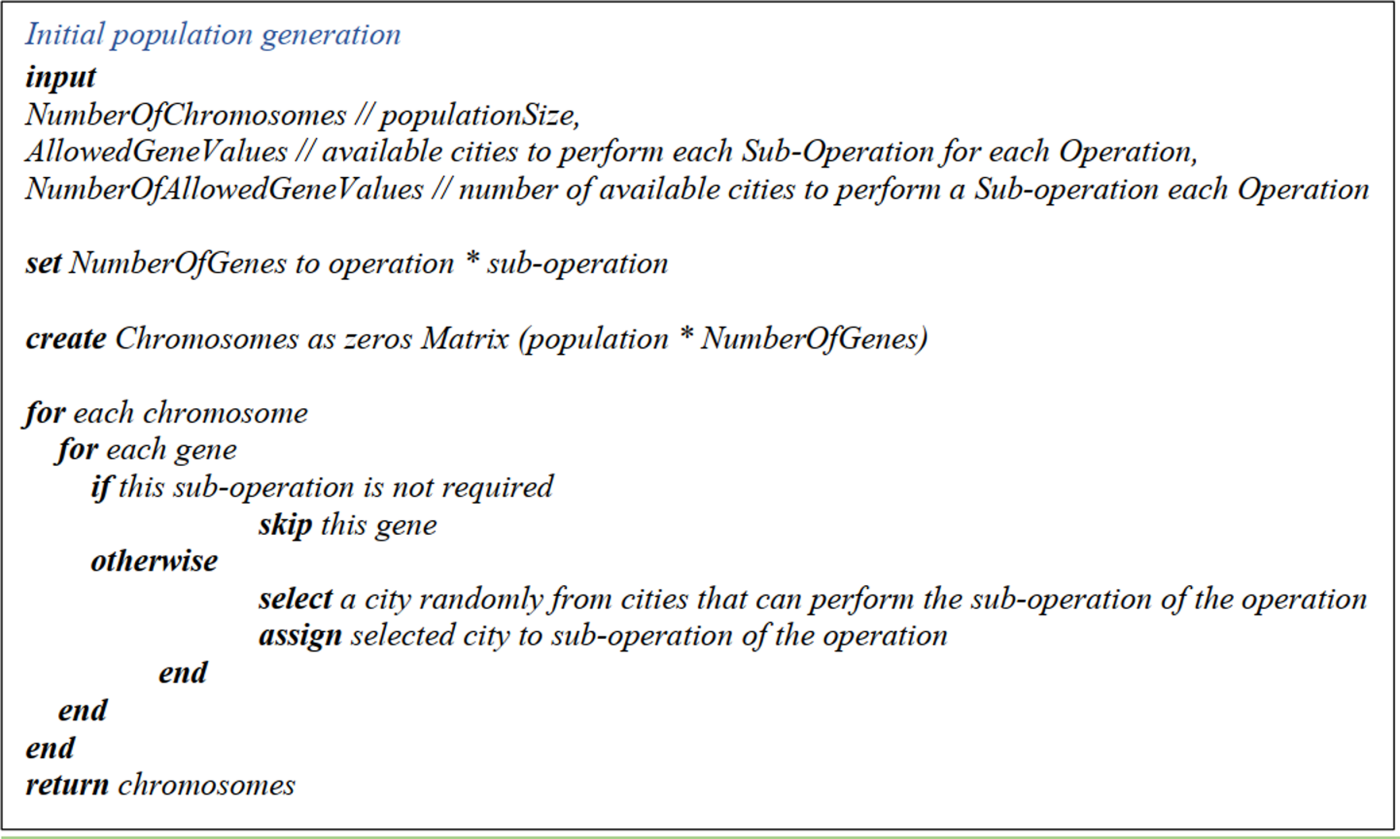
The pseudo-code of the initial population generation.

Then, for each gene in the matrix, if the respective sub-operation is required for a specific operation, the number of the cities which supply the specified sub-operation of this operation is derived from the Number of Allowed Gene Values Matrix. Next, a city is randomly picked from the corresponding column (sub-operation of the specified operation) from the Allowed Gene Values Matrix. This procedure is repeated for the whole matrix. The pseudo-code is displayed in Fig. 4.

##### Chromosome cost calculation

**Figure 5:**
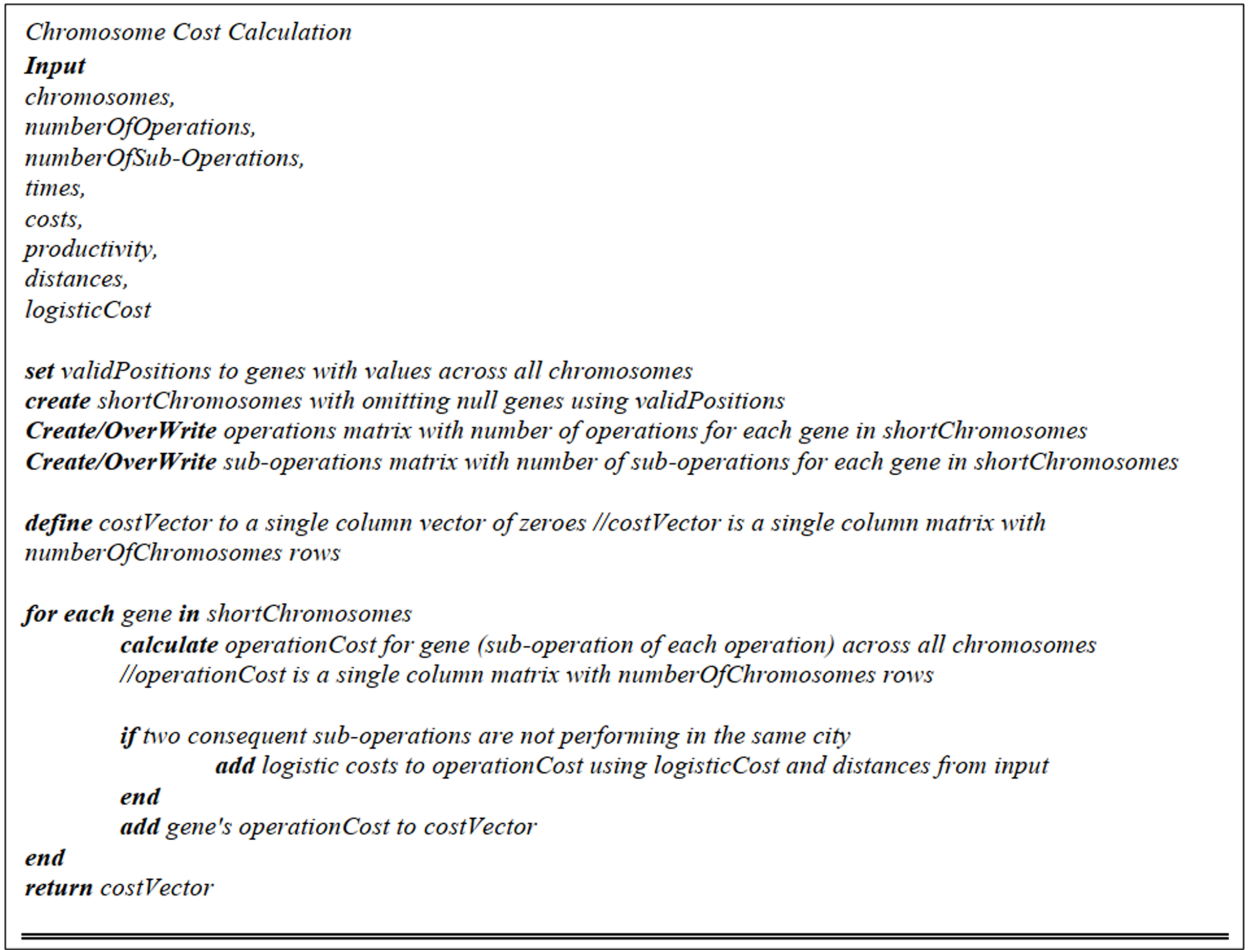
Chromosome cost calculation pseudo-code.

After the cities are assigned to the required sub-operations, the costs have to be calculated by multiplying the sub-operation performing time in a particular city by the sub-operation performing cost, and then divided by the service quality in that city (service point). The pseudo-code is displayed in Fig. 5.

Likewise, the transportation service cost is yielded by multiplying the distance between the specified cities (service points) by a presumed expense, a constant currency unit per mile (in this study, it is 0.3). Ultimately, each chromosome's total cost, which is the sum of performing costs and transportation costs, is calculated and saved in the Final Cost Matrix.

**Cross over**

##### Selection probability matrix

Amongst the various approaches of selecting parents from chromosomes, we preferred a rank-based selection method. The Selection Probability Matrix is a one-column matrix, with the rows as many as the population number that contains digit one in each cell. After that, the selection probability of each chromosome is yielded in the following manner:

First, each chromosome’s rank is identified regarding the cost calculated and saved in the Final Cost Matrix. Secondly, a weight factor, between zero and one, is powered to the chromosome’s rank. Eventually, all the yielded values are divided by the total sum of all the matrix’s calculated values, which results in normalized values less than one.

##### Accumulative selection probability

**Figure 6:**
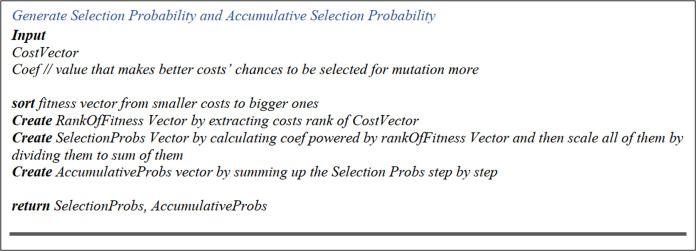
Selection probability and accumulative selection probability pseudo-code.

In this matrix, to work out each cell’s value (chromosome), from Selection Probability Matrix, all prior cells’ selection probabilities are added up and stored in this cell. Therefore, the first cell’s content is the selection probability of the first element of the Selection Probability Matrix. Figure 6 displays the pseudo-code of “Selection Probability Matrix” and “Accumulative Selection Probability”.

**Rank-based selection**

##### Selected indexes matrix and parents matrices

**Figure 7:**
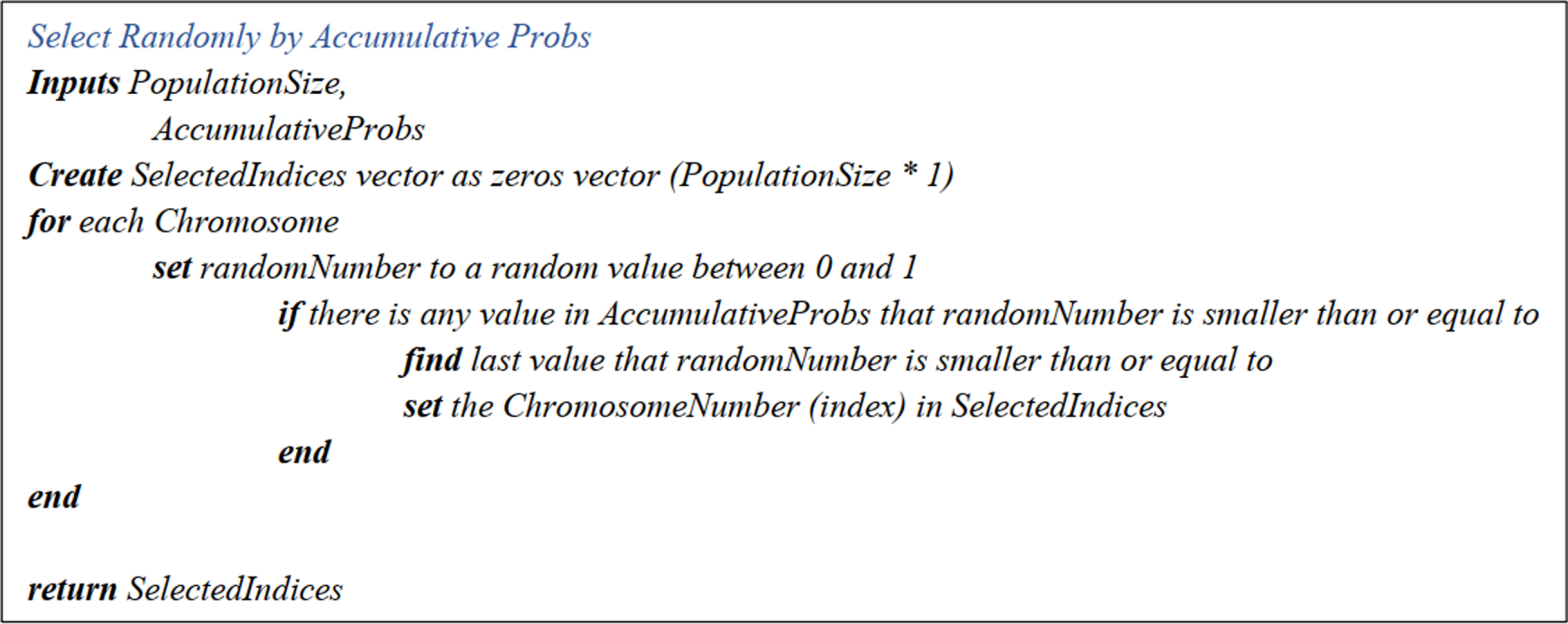
Select randomly by accumulative probs pseudo-code.

The Uniform Crossover function exploited in this research is carried out as follows. In the beginning, the Selected Indexes Matrix with only one column, and the number of the rows equal to the population size is generated. Initially, all the values are set zero. Secondly, the population size is derived based on randomly generated figures between zero and one. Next, the Accumulative Selection Probability Matrix is referred to; as soon as a row whose number is greater than the randomly generated number, the row number is put in the matrix, respectively. Lastly, the chromosomes are rearranged according to these indexes, forming the Parents Matrix, wherein the chromosomes with even indexes are known as the first parent, while the odd ones form the second parent. The pseudo-code is illustrated in Fig. 7.

##### Uniform cross over, cross over mask and offspring matrices

**Figure 8:**
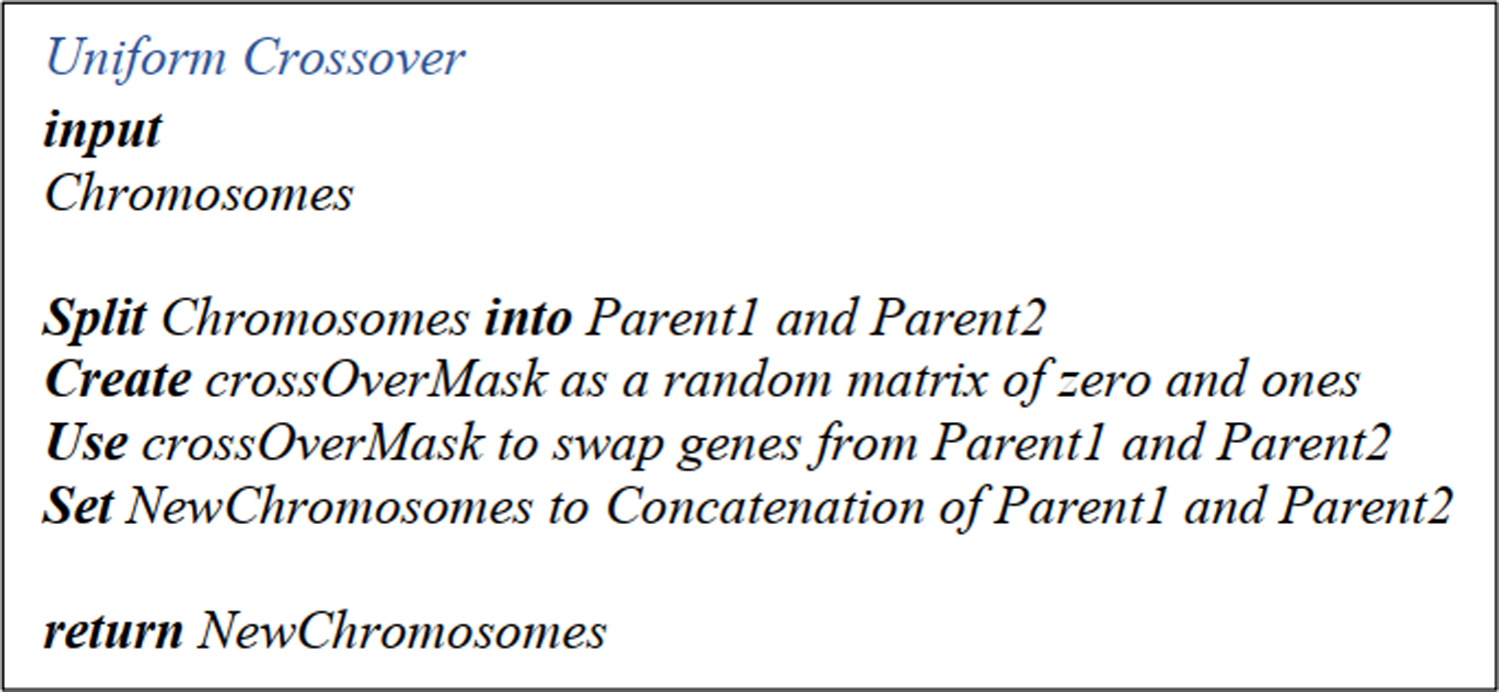
Uniform CrossOver pseudo-code.

After creating the Parents Matrixes, the next step is building up a Crossover Matrix, wherein the number of rows equates to half the number of population and columns equals the number of operations multiplied by the sub-operations. This matrix only contains ones and zeros, which are randomly put in the cells. Then the offsprings are generated in the following manner. Firstly, the first offspring receives the second parent's chromosomes, while the second offspring receives all the first parent's chromosomes. Next, the Crossover Matrix genes are once modified for the first offspring and then for the second one. The procedure is as follows: the corresponding row in the Crossover Matrix is checked; if it is zero, then the current value is kept; if it is one, it takes the other parent's gene’s value. Finally, both offsprings rewritten in the Chromosomes Matrix are subjected to mutation. The pseudo-code is shown in Fig. 8.

##### Mutation, mutation mask, mutant chromosomes matrix

**Figure 9:**
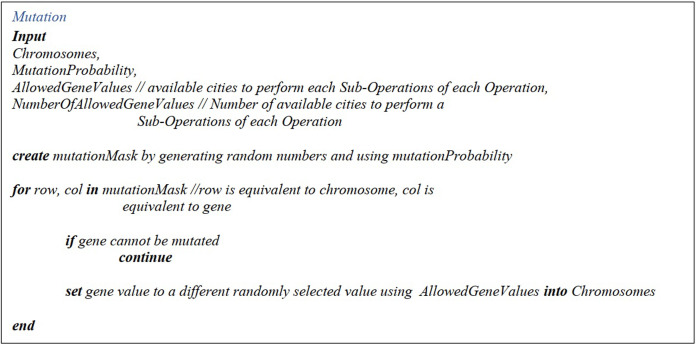
Mutation pseudo-code.

After the Mutation Mask Matrix is generated, with the population size as the number of rows and the number of operations timed the number of sub-operations as the number of the columns, it is filled with randomly generated figures from zero to one. Afterward, the random values greater than the mutation parameter are set zero, or else are put one. Afterward, each gene is checked out. If the respective figure in the Number of Allowed Genes Matrix is greater than one, it will be modified. A figure from the allowed values column of the Allowed Genes Matrix values substitutes it; otherwise, the value is maintained. The pseudo-code could be found in Fig. 9.

##### Seeking the best chromosome

After the Genetic Algorithm is thoroughly executed, the solution associating with the minimum cost is considered the best solution (yielded by the algorithm). As is explained in “Initial Population Generation”, this chromosome is now a single-rowed matrix whose columns are equal to the product of operations by sub-operations. This chromosome is later reshaped into the Operations Matrix as clarified in “Operation/Sub-Operation” and “Operations”.

##### Calculation of fulfilled sub-operations, updating the operations matrix, finished operations matrix, list of the finished operations of each run

When a chromosome is reshaped into the form of the Operations Matrix, the sub-operations are examined in the specified time interval to see if their scheduling was completed or still in progress. Thus, the performing time of each operation’s sub-operation is extracted from the Time Matrix and then subtracted from the specified time interval one after another until the result turns smaller than zero, which means there is no time left for any other sub-operations. Next, each of the operations whose scheduling is completed in the interval is set zero in the Operations Matrix; in other words, they are eliminated from the Operations Matrix.

Furthermore, all the finished sub-operations in each time interval (at each run) are stored in a matrix named Finished Operation Matrix. The dimensions of this matrix are like the new version of the Operation Matrix and are initially filled with zeros. When the Operations Matrix is filled with the finished sub-operations, the matrix generated at each run is saved in the Finished operations’ List.

##### Appending new operations to the existing ones

After updating the Operations Matrix, the new operations are simply appended to the end of the Operations Matrix. Since synthetic data is used in this study, recent operations are generated and appended to the matrix, as described in this section.

##### Calculation of the number of fulfilled operations and the total cost

The different stages of the algorithm will keep running until the stopping condition is met. The scheduled operations’ costs are worked out according to the Chromosome Cost Calculation after the algorithm stops running. The number of scheduled operations and sub-operations will also be extracted from the Finished operations’ List.

## Illustrative case study

As was mentioned earlier, one of the main concerns of the present study is solving dynamic, large-sized service composition problems, including logistics, maintaining the Quality of Service at a desirable level. Thus, in this chapter, the results yielded by exploiting the improvised algorithm (mentioned in “The Presented Algorithm for Solving the Model Considering Runtime and Dynamicity”) are illustrated and discussed.

First, in “Assessment of the Genetic Algorithm Performance”, the genetic algorithm's performance as the main core of the Dynamic Service Composition Algorithm is explicated. Next, in “The Performance Assessment of the Dynamic Service Composition Algorithm”, the performance of the Dynamic Service Composition Algorithm is assessed.

### Assessment of the genetic algorithm performance

**Figure 10:**
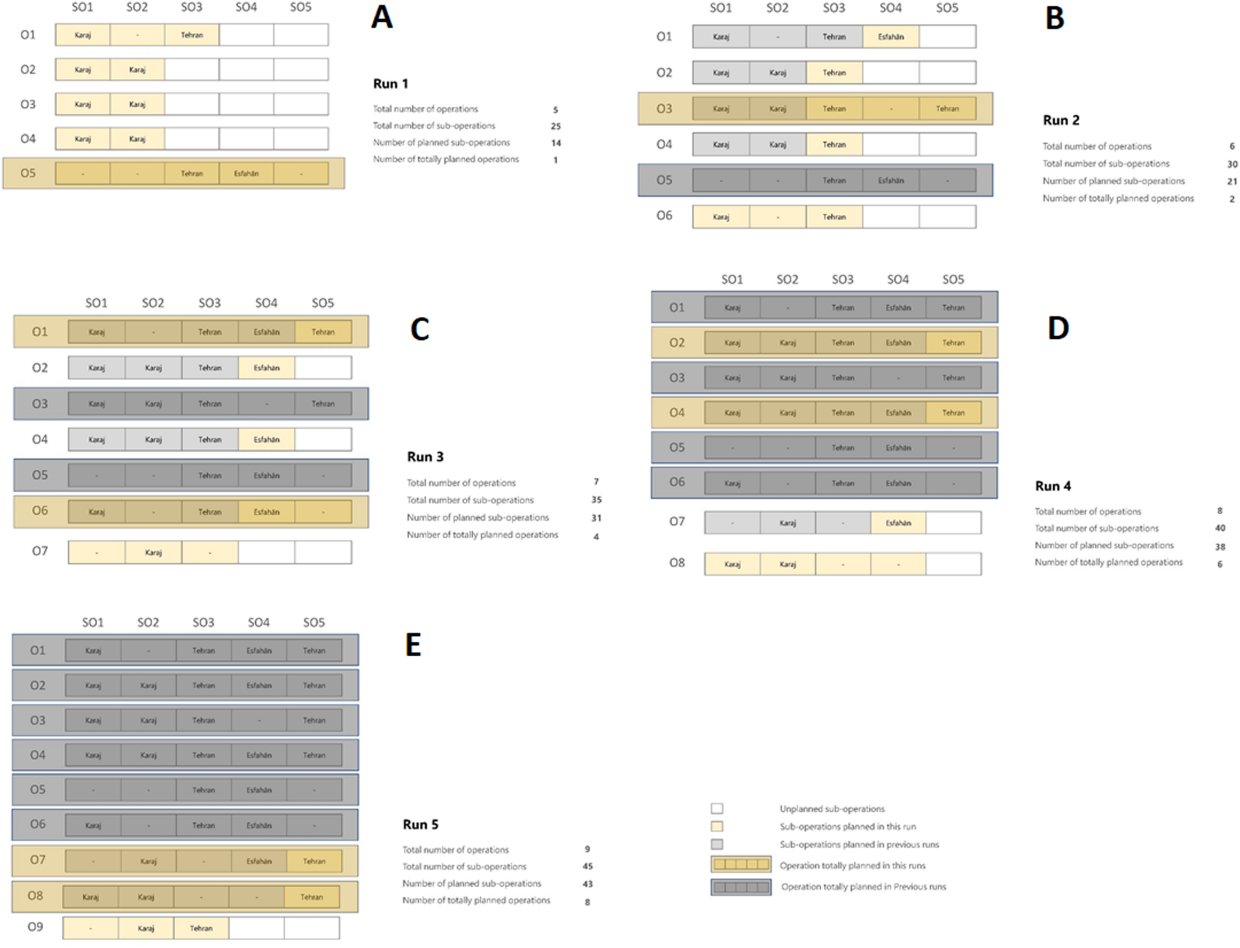
The depiction of the sample problem solved by the developed algorithm (Number of Operations, Number of Sub-operations, Number of planned Sub-operations, Number of Totally planned operations). (A) 5,25,14,1; (B) 6,30,21,2; (C) 7,35,31,4; (D) 8,40,38,6; (E) 9,45,43,8.

The synthetic data for operations and sub-operations is randomly generated according to “Distance Matrix” to “Productivity Matrix” that is available through the repository at DOI 10.6084/m9.figshare.14229299.v1 to solve the sample problems. In order to solve the large-sized problems, which is the main contribution of this paper, a number of cities across the country (Iran) are considered as the service points. The Euclidian distance between pairs of cities calculated using the geographical coordinates is regarded as the distance between the service points and stored in the Distance matrix. A sample problem of five different operations, each consisting of 20 sub-operations and 20 cities as service-points, is demonstrated in Fig. 10.

Furthermore, to solve such problems by the Genetic Algorithm, the main parameters, namely the initial population, the number of generations, the cross over influence coefficient, and the mutation rate, have to be determined. Moreover, each of these parameters has to be optimized according to the size of the problem. In this research, the Taguchi method was exploited to yield the parameters’ optimal values, which resulted in the following values:

1. The size of the population is set 720 chromosomes.
2. The crossover influence coefficient is assumed to be 0.98.
3. The mutation rate: this parameter is supposed to enhance the algorithm's performance by exploring the unchecked areas of the solution space; a high rate of mutation may result in unwanted random explore. Hence, a value as small as 0.002 is selected as the rate of mutation in this study.
4. The number of iterations is the stopping condition of the Genetic Algorithm, which in this example is set to 90. The Genetic Algorithm would end after completing 90 iterations.

**Figure 11:**
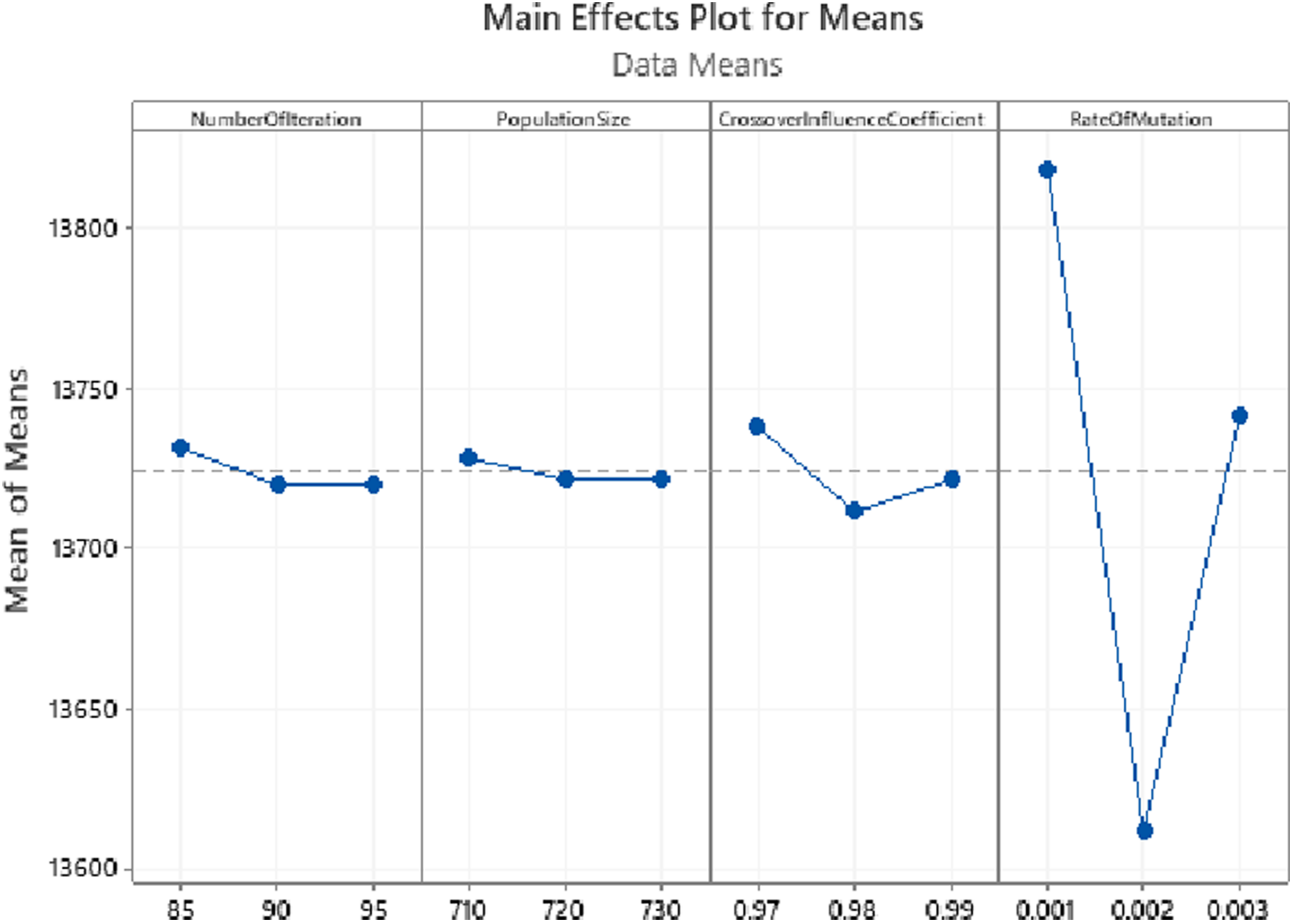
Parameter tuning using Taguchi method for the synthetic problem with five operations, 20 sub-operations for each operation, and 20 cities.

**Figure 12:**
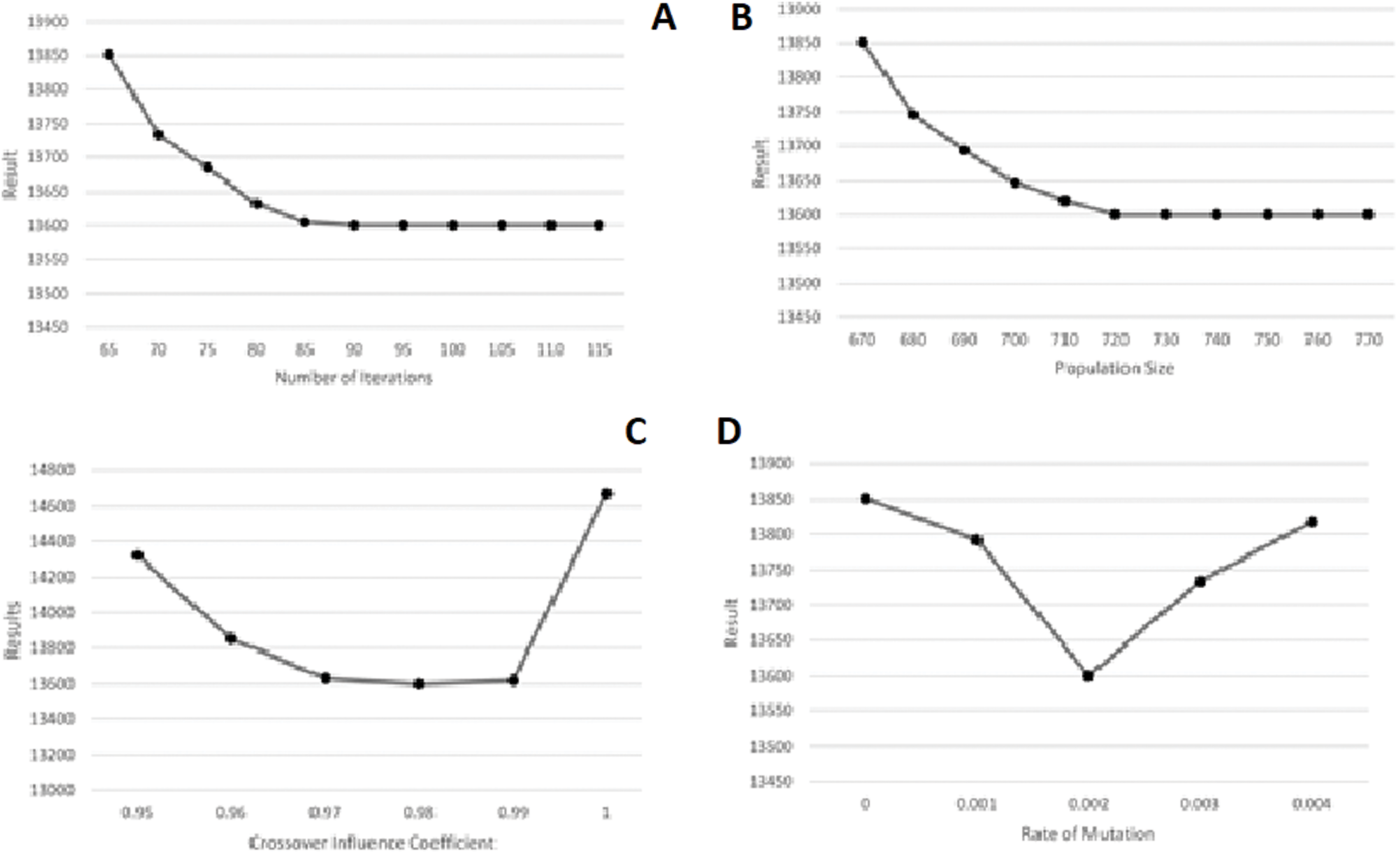
Parameter tuning with loops for the synthetic problem with five operations, 20 sub-operations for each operation and 20 cities. (A) Number of Iterations. (B) Population Size. (C) Crossover Influence Coefficient. (D) Rate of Mutation.

The Taguchi approach utilized Minitab 19 Statistical Software. According to Minitab’s Instructions for the design of experiments in the Taguchi method, three levels -shown in Fig. 11—were taken for each parameter. Regarding the experiments designed in Minitab and the values taken by the software, GA was executed, and the resulting values for total costs were inserted into the software as their responses. The parameters’ optimal values yielded this way are demonstrated in Fig. 11. As could also be observed in Table 3, the parameters produced by the Minitab software are listed from the most to the least sensitive to change as following: RateOfMutation, CrossoverInfluenceCoefficient, NumberOfIterations, and PopulationSize. Finally, as illustrated in Fig. 11, the results are almost stable for NumberOfIteration and PopulationSize after reaching their optimal values, showing very little change. The GA algorithm was executed with higher values for the two latter parameters to ensure that these two parameters’ yielded values are optimal. The results were still stable, as could be seen in Fig. 12.

**Table 3:** Taguchi response table for means.

Level	Number of iteration	Population size	Crossover influence coefficient	Rate of mutation
1	13,732	13,728	13,738	13,818
2	13,720	13,722	13,712	13,612
3	13,720	13,722	13,722	13,742
Delta	12	7	27	207
Rank	3	4	2	1

Each sample problem presented in this section has once been resolved by the software LINGO 18.0, and secondly, by the improvised algorithm of this study utilizing MATLAB 2017b programming engine. All the executions were performed on a Windows 10 × 64 -operated computer with an Intel Core i5 CPU and a RAM of 8 GB.

Three sets of synthetic data for operations/sub-operations were randomly created, as mentioned before, to assess the precision and speed of the Genetic Algorithm. The hardship of the problems increases in an ascending manner from problem no.1 to no.3.

The average outcomes for ten runs of each sample problem are summarized in Table 4. In Table 4, the ‘Problem’ column indicates the problem number. The ’Operation’, ‘Sub-operation’, ‘City’ column respectively show the number of operations, the number of sub-operations of each operation, and the number of the cities serving as service points. Also, the contents of the ‘optimal-S’ column are the optimal values yielded by LINGO, where the ‘estimated-S’ holds the values calculated by the Genetic Algorithm for the objective function. The differences between the above values are stored in the ‘error’ column. The ‘error portion’ column indicates the ratio of the ‘error‘ contents divided by the optimal values obtained by LINGO. The ‘optimization-T’ column has the LINGO performing time consumed to yield the optimal solution, as long as the ‘estimation-T’ exhibits the time the presented Genetic Algorithm took to achieve the optimal or near-optimal solution. Lastly, the ‘time portion’ column indicates the ratio of the performing times of the two methods. (LINGO performing time to the presented Genetic Algorithm).

**Table 4:** The comparison between the solution obtained by the developed genetic algorithm and the exact solution.

Operation, sub-Operation, City	Problem	Optimal-S	Estimated-S	Error	Error portion (%)	Optimization-T	Estimation-T	Time portion
5, 5, 5	1	2,648.63	2,648.63	0.00	0	0.28	0.03	10.73
	2	5,944.42	5,944.42	0.00	0	0.35	0.03	11.67
	3	6,653.88	6,653.88	0.00	0	0.4	0.04	10.8
5, 10, 10	1	5,086.08	5,145.03	58.95	1.16	31	0.34	104.47
	2	7,352.66	7,409.04	56.38	0.77	72	0.09	774.91
	3	7,652.32	7,714.85	62.53	0.82	88.50	0.18	824.63
10, 10, 10	1	11,999.47	12,656.17	656.70	5.47	202.44	0.23	876.62
	2	14,248.72	14,322.24	73.52	0.52	272.87	0.23	1,197.53
	3	14,200.75	14,303.49	102.7	0.72	315.21	0.26	1,229.19
5, 10, 20	1	4,252.04	4,289.83	37.79	0.89	344.37	0.11	3.260.95
	2	5,497.294	5,543.21	45.91	0.84	968.98	0.13	7.458.84
	3	5,866.17	5,925.18	59.00	1.01	1,657.37	0.14	1,2370.32
5, 20, 10	1	13,512.96	13,805.60	292.64	2.17	326.92	0.40	814.79
	2	15,803.26	16,137.08	333.82	2.11	342.99	0.36	952.62
	3	16,573.11	17,243.44	670.33	4.04	707.85	0.56	1,299.41
5, 20, 20	1	11,392.82	11,875.55	482.7	4.24	4,949.27	0.55	9,038.46
	2	13,045.16	13,580.50	535.34	4.10	10,389.29	0.71	15,005.82
	3	14,366.02	14,899.99	533.97	3.72	72,934.85	0.71	106,263.78

Apparently, from the results, the solution obtained by the developed Genetic Algorithm presented by this study is near-optimal by an acceptable tolerance, and the problem is solved remarkably faster than LINGO. As a matter of fact, the optimum solution yielded by the presented algorithm is almost instant, which means the algorithm overcomes the classical approaches by distinction.

### The performance assessment of the dynamic service composition algorithm

In addition, to compare the two approaches clearly, the performing time of LINGO to find the optimal solution was defined as the basis of the comparison so that this performing time is set as the stopping condition for the proposed Dynamic Service Composition Algorithm.

A problem of five operations, five sub-operations for each operation, and five cities (service points) are assumed for simplicity. This problem has a medium level of complexity among the three aforementioned problems (problem number two). So, the algorithm performing time is 0.35 s, and the scheduling time interval is considered ten units of time. The results yielded from the algorithm's execution at each run are demonstrated in Figs. 10A–10E. The algorithm started with five operations, then at each run, one operation was added to the previous ones. In Fig. 10, the rows represent the operations, and the columns indicate sub-operations. Moreover, the golden boxes depict the scheduled sub-operations in the current time interval. The white boxes show the unplanned sub-operations in the current time interval (the sub-operations that exceed the time interval), as long as the grey boxes exhibit the sub-operations planned in the previous time intervals. In addition, if an operation’s scheduling was finished in the current time interval, a golden box, and providing that the whole operation’s scheduling was completed in the previous runs, a grey box covers the whole sub-operations of that operation. As manifested in Fig. 10, the algorithm was executed five times and finally completely scheduled eight operations out of nine and 43 sub-operations out of 45 in the specified time interval.

Moreover, as Table 4 demonstrates, the optimal solution of this problem in the static mode costs 5,944.42 units of currency; in contrast, solving the same problem in a similar time interval in dynamic mode costs 8,277.89 units of currency; nevertheless, 18 sub-operations more than the static mode were scheduled. So, the cost per sub-operation for LINGO is 237.7768, where it is 192.5091 for the proposed GA, which means that LINGO is 1.24 times costlier, as shown in the column ‘LINGO to Alg. Cost Portion’. In other words, as long as a part of the cost forms the income of the service providers, it is claimed that the proposed algorithm not only handled a greater number of the requests in a similar time interval, which is one of the satisfaction factors of the clients but also could create higher revenue. A comparison between the algorithm's outcomes using LINGO in the static mode and the developed algorithm in dynamic mode is presented in Table 5. All the comparisons are carried out on a problem with a medium level of complexity.

**Table 5:** The comparison between the static and dynamic algorithms.

Operation, Sub-operation, city	LINGO total cost	Alg. operations done	Alg. sub-operations done	Alg. total cost	Operation portion	Sub-operation portion	LINGO cost per sub-operation	Alg. cost per sub-operation	LINGO to Alg. cost portion	LINGO Sub-operation Loss	Alg. Total Operations	Alg. Portion of Done Operations
5, 5, 5	5,944.42	8	43	8,277.89	1.6	1.72	237.7768	192.5091	1.24	18	9	88.89%
5, 10, 10	7,352.66	135	1,228	176,195.28	27	24.56	147.0532	143.4815	1.02	130	140	96.42%
10, 10, 10	11,999.47	251	2,517	287,260.77	25.1	25.17	119.9947	114.1282	1.05	2,417	254	98.82%
5, 10, 20	5,497.29	489	4,896	464,214.64	97.8	97.92	109.9458	94.8151	1.16	4,846	491	99.59%
5, 20, 10	16,137.08	200	4,012	520,010.90	40	40.12	161.3708	129.6139	1.25	3,912	205	97.56%

In Table 5, the ‘Operation’, ‘Sub-operation’ and ‘City’ respectively indicate the number of operations, sub-operations, and cities. The ‘LINGO Total Cost’ column contains the optimal cost of performing services reported by LINGO. The ‘Alg. Operations Done’, ‘Alg. Sub-operations Done’ and ‘Alg. Total Cost’ show the number of the operations wholly scheduled, the number of the sub-operations scheduled, and the sub-operations' performing cost in the LINGO performing time for the size of the problem specified in column ‘Operation, Sub-operation, City’, respectively. In columns ‘Operation Portion’ and ‘Sub-operation Portion’, the proportion of wholly scheduled operations by the proposed GA to the LINGO’s number of the operations and the proportion of scheduled sub-operations by the proposed GA to the LINGO’s total number of sub-operations are demonstrated respectively. The ‘LINGO Cost Per Sub-operation’ and ‘Alg. Cost Per Sub-operation’ columns represent the performance cost per each sub-operation for LINGO and the proposed GA, respectively, where the ‘LINGO to Alg. Cost Portion’ shows the proportion of the two mentioned columns. The ‘LINGO Sub-operation Loss’, and ‘Alg. Total Operations’ contain the number of the sub-operations that were not received or scheduled by LINGO, and the total number of the operations received by the dynamic algorithm, respectively. Finally, the ‘Alg. Portion of Done Operations’ column shows the proportion of wholly scheduled operations to the total number of the operations received by the dynamic algorithm. According to Table 5, the developed dynamic algorithm shows better performance, and in all the samples achieved remarkably better performance and revenue.

## Conclusion

In recent years, manufacturing industries have been encouraged to deploy Information Technology paradigms to survive the rivalry circumstances of manufacturing competitive environments. Industry 4.0 and Cloud manufacturing, accompanied by a service-oriented architecture model, have been regarded as the most recent IT paradigms that enable and facilitate conventional manufacturing models' transition into more efficient and productive digitalized models. The XaaS (Everything as a Service), based on a decentralized manufacturing focus, enables clients to acquire required services and resources in digitalized clouds. These clouds consist of geographically distributed manufacturing and logistics service providers, which can be reached through modern IT capabilities such as IoT. These services can be composed to fulfill the complex requirements of manufacturing and logistics services. Therefore, efficient service composition is a remarkable topic in Cloud manufacturing due to the large size and operational complications, mainly caused by a large number of continually received service requests having to be prioritized and handled in the minimum possible time, observing the quality of the composing service. Heuristic and metaheuristic solving approaches are strongly preferred to obtain optimal or nearly optimal solutions considering the NP-hard nature and dynamicity of the allocation problem in the cloud.

This study has presented an innovative, time-efficient approach for dynamic manufacturing and logistics service composition with the QoS considerations. The method presented in this paper has applied a heuristic approach by considering the interdependencies among manufacturing and logistics services. It has modified the evolution phase in solving the service composition by tuning the generation of new solution alternatives. The results have shown that the proposed algorithm is highly competent in solving large-scale problems dynamically, efficiently, almost instantly, with sufficient optimality. The present research also has proposed an algorithm to treat the dynamic behavior of manufacturing and logistics services. It enables the service composition model to consider the addition of new services and demands to service pools. The proposed model adopts the service composition model to include the newly introduced demands and fulfill them through the current service composition solution. This capability has not been approached in the literature of service composition models. However, it is necessary for the XaaS framework, since the arrival of new service or demand instances is inevitable and should be considered through the service composition algorithm. Future research studies are strongly recommended to consider the other dynamic events in the manufacturing service composition problem like the disruptions in service fulfillment. Also, expanding the QoS function to consider the midterm decision-making criteria like reliability and reputation is strongly recommended. The research studies for modeling cloud manufacturing scenarios of dynamic behaviors of service providers and demanders, considering the new insight proposed in this paper to fulfill the dynamic ecosystem of service composition, especially by applying reinforcement learning concepts are strongly proposed.

## Additional Information and Declarations

### Data Availability

The following information was supplied regarding data availability: Data is available at Figshare: Fatahi Valilai, Omid (2021): DataSet for logistics and manufacturing service composition. figshare. Dataset. DOI 10.6084/m9.figshare.14229299.v1. Codes are also available at Figshare: Fatahi Valilai, Omid (2020): Dynamic Mutual Manufacturing and Transportation Routing service. figshare. Software. DOI 10.6084/m9.figshare.13173218.v1.
